# Melatonin Sensitizes Human Colorectal Cancer Cells to γ-ray Ionizing Radiation In Vitro and In Vivo

**DOI:** 10.3390/ijms19123974

**Published:** 2018-12-10

**Authors:** Qin Wang, Zhijuan Sun, Liqing Du, Chang Xu, Yan Wang, Bing Yang, Ningning He, Jinhan Wang, Kaihua Ji, Yang Liu, Qiang Liu

**Affiliations:** 1 Tianjin Key Lab of Radiation Medicine Molecular Nuclear Medicine, Institute of Radiation Medicine, Chinese Academy of Medical Science and Peking Union Medical College, Tianjin 300192, China; wangqin@irm-cams.ac.cn (Q.W.); sunzj@irm-cams.ac.cn (Z.S.); dlq@irm-cams.ac.cn (L.D.); xuchang@irm-cams.ac.cn (C.X.); wangyan@irm-cams.ac.cn (Y.W.); heningning@irm-cams.ac.cn (N.H.); wangjinhan@irm-cams.ac.cn (J.W.); jikaihua@irm-cams.ac.cn (K.J.); 2 Department of Cellular Biology, School of Basic Medical Sciences, Tianjin Medical University, Tianjin 300070, China; yangbingtj@aol.com

**Keywords:** melatonin, colorectal cancer, ionizing radiation, radiosensitivity

## Abstract

Colorectal cancer is the most commonly reported gastrointestinal malignancy, with a recent, rapid increase of the annual incidence all over the world. Enhancing the radiosensitivity of cancer cells while preserving the health of normal cells is one of the most important tasks in clinical radiobiology. However, resistance to radiotherapy for colorectal cancer greatly decreases the therapeutic outcome. Melatonin (*N*-acetyl-5-methoxytryptamine), a natural secretory product that the pineal gland in the brain normally produces, has been reported to have anticancer properties. In the study, we investigated the combination of melatonin with radiotherapy as a treatment for colorectal cancer. We firstly explored the anti-tumor activity of melatonin combined with ionizing radiation (IR) against colorectal carcinoma in vitro. It was found that melatonin effectively inhibited human colorectal carcinoma cell line HCT 116 cellular proliferation, colony formation rate and cell migration counts following IR. Increasing the radiosensitivity of colorectal cancer cells by melatonin treatment was found to be associated with cell cycle arrest in the G2/M phase, downregulation of proteins involved in DNA double-strand break repair and activation of the caspase-dependent apoptotic pathway. Moreover, we also investigated the combined effect of IR and melatonin on colorectal tumor in vivo. Results from a tumor xenograft showed that melatonin plus IR treatment significantly suppressed tumor cell growth compared with melatonin or IR alone, resulting in a much higher tumor inhibition rate for the combined treatment. The data suggested that melatonin combined with IR could improve the radiosensitivity of colorectal cancer and thus enhance the therapeutic effect of the patients, implying melatonin could function as a potential sensitizer in tumor radiotherapy.

## 1. Introduction

Colorectal cancer is one of the main causes of cancer death worldwide. It is reported that colorectal cancer is the third most commonly diagnosed cancer and the second most frequent cause of cancer-related death, with over 1.8 million new cancer cases and 881,000 deaths having occurred in 2018 [1]. Clinically, surgery is considered as the main therapeutic measure for colorectal cancer, combined with either radiotherapy alone or a combination of radiotherapy and chemotherapy. Radiotherapy is considered as an effective treatment option after surgery. Unfortunately, colorectal cancer exhibits resistance to ionizing radiation (IR) in radiation oncology treatment. A high dose of radiation that is required to be delivered to the tumor results in the damage to adjacent normal tissues or organs. Limited alternatives in therapeutic strategies and resistance to IR treatment compose the challenges of improving the survival of colorectal cancer patients. Therefore, it is urgent to develop additional novel therapeutic alternatives for colorectal carcinoma.

Melatonin (*N*-acetyl-5-methoxytryptamine), discovered by A. Lerner in 1958 [2], is a main hormone that the pineal gland in the brain of mammals and humans normally produces. Initially, melatonin was found to be involved in the control of circadian rhythms of diurnal species [3]. Actually, melatonin is reported to participate in the regulation of a variety of physiological and pathological processes, including antioxidation, anti-aging, anti-inflammation, anti-angiogenesis, stimulation of cell differentiation, and activation of the immune system [4,5]. In recent years, numerous experimental studies have revealed that melatonin exerts an antitumour effect in different types of cancer in vitro, such as breast cancer [6], lung carcinoma [7], esophageal cancer [8], pancreatic cancer [9], and prostate cancer [10]. In addition, melatonin treatment on animals with tumor xenografts inhibited tumorigenesis [11,12,13].

Studies have found that melatonin exhibits antitumor potency in different colorectal cancers [14,15,16,17,18]. Melatonin’s oncostatic effect was related with its antioxidative and anti-inflammatory functions, neutralizing the oxidative stress and suppressing the nitric oxide production of colorectal cancer cells [19]. In clinical trials, melatonin showed the ability to enhance the therapeutic efficacy of other chemotherapeutic drugs, as shown by induced tumor regression, high survival rate and relieved symptoms of side effects in patients with metastatic colorectal cancer [20]. Collectively, melatonin may be an appealing therapeutic strategy for colorectal cancer.

It is worth noting that studies involving the use of melatonin as a radiosensitizer have shown promising results. Melatonin has a synergic effect on the therapeutic outcomes of chemotherapy and radiotherapy on head, neck and liver cancer cells [21,22]. Zou et al. [23] found that melatonin synergized with radiation could induce cytotoxicity to thyroid carcinoma. Melatonin pre-treatment prior to IR induced an increase in the radiosensitivity of breast cancer cells and a decrease in radiation-induced side effects in both rodent models and breast cancer patients [6,24,25]. However, few studies have been undertaken to investigate the combination of melatonin with radiotherapy as a treatment for colorectal cancer. In addition, the effects and therapeutic potential of melatonin treatment prior to radiation on colorectal carcinoma remain to be explored. In this study, we examined the effects of melatonin on cell growth and sensitization to γ- ray radiation in colorectal cancer in vitro. Further, we investigated whether melatonin could inhibit the tumor growth of tumor-bearing nude mice exposed to IR in vivo.

## 2. Results

### 2.1. Effects of Melatonin on the Proliferation of HCT 116 Cells

**Figure 1:**
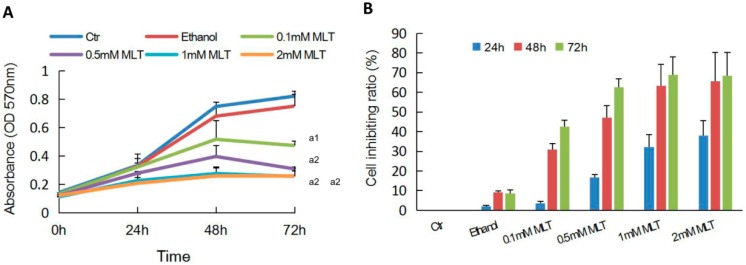
Melatonin inhibited the proliferation of human colorectal carcinoma cell line HCT 116 cells. (**A**) HCT 116 cells were treated for 0, 24, 48 or 72 h with various concentrations of melatonin (0.1, 0.5, 1, and 2 mM) or vehicle (0.1% ethanol). Absorbance at OD 570 nm was measured by MTT assay; (**B**) the inhibition ratio of HCT 116 cells exposed to various concentrations of melatonin after 24, 48 or 72 h of treatment was detected. The cell inhibition ratio = (1 − OD value_treated group_/OD value_control group_) × 100%. Data are presented as the mean ± SD. ^a1^ *p* < 0.05; ^a2^ *p* < 0.01 vs. control.

In the study, we firstly used human colorectal carcinoma cell line HCT 116 to evaluate the effect of melatonin alone on cell proliferation. As shown in Figure 1A, cells of the control group and ethanol group grew steadily as shown by the linear shape. Whereas, cells treated with various concentrations of melatonin (0.1, 0.5, 1 and 2 mM) stepped down their growth after 48 h of treatment. Melatonin suppressed cell viability of HCT 116 cells in a time-dependent and dose-dependent manner (Figure 1B). As incubation with 1 mM or 2 mM melatonin induced the similar effect on HCT 116 cell proliferation, we selected 1 mM concentration of melatonin as the dose in the following experiment.

### 2.2. Effects of Melatonin on Colony Formation and Migration of HCT 116 Cells Induced by Radiation

**Figure 2:**
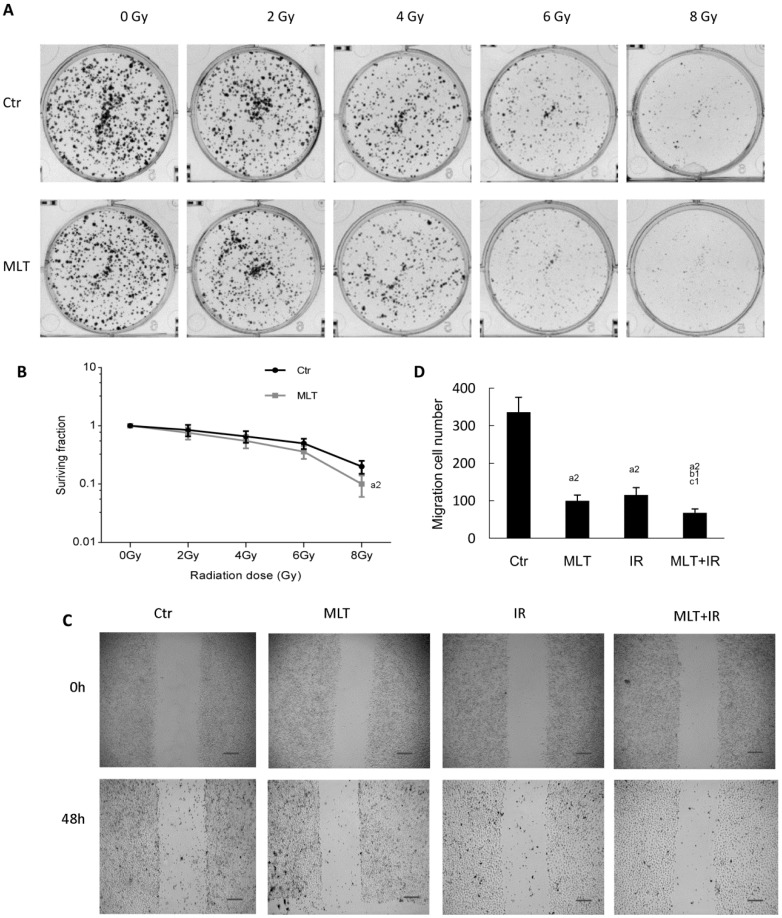
Melatonin suppressed the colony formation and migration of HCT 116 cells exposed to γ-ray radiation. (**A**) HCT 116 cells were treated with or without 1 mM melatonin for 2 h, then exposed to the indicated dose of γ-ray radiation of 0, 2, 4, 6, or 8 Gy, and cultured for 2 weeks. Representative images of colony formation are displayed; (**B**) A minimum of 50 viable cells were scored as a colony. The surviving fraction was calculated; (**C**) HCT 116 cells were treated with or without 1 mM melatonin for 2 h, then exposed to 6 Gy γ-ray radiation or not. Representative images of HCT 116 cell migration at different time points (0 and 48 h) are displayed, scale bar, 100 μm; (**D**) the migration cell count at 48 h was calculated by analyzing five fields/sample. Data are presented as the mean ± SD. ^a2^ *p* < 0.01 vs. control, ^b1^ *p* < 0.05 vs. IR, ^c1^ *p* < 0.05 vs. MLT.

Next, we explored the sensitivity of HCT 116 cells treated with melatonin following radiation. We performed the colony formation assays of cells exposed to 0, 2, 4, 6, or 8 Gy radiation. It was observed that melatonin pretreatment induced a significant decline in the colony count in HCT 116 cells compared with the control (Figure 2A), and the clonogenic survival curve showed that the melatonin line was obviously more sensitive to radiation than the control line (Figure 2B).

Moreover, we assessed the influence of melatonin on cell migration. As shown in Figure 2D, melatonin or IR dramatically reduced HCT 116 cell migration, and melatonin plus IR induced a statistically significant reduction in cell migration compared to melatonin or IR alone. Given all this, it should lead to the conclusion that melatonin increased the sensitivity of HCT 116 cells to IR in vitro.

### 2.3. Effect of Melatonin on Cell Cycle and Cell Apoptosis of HCT 116 Cells Induced by Radiation

**Figure 3:**
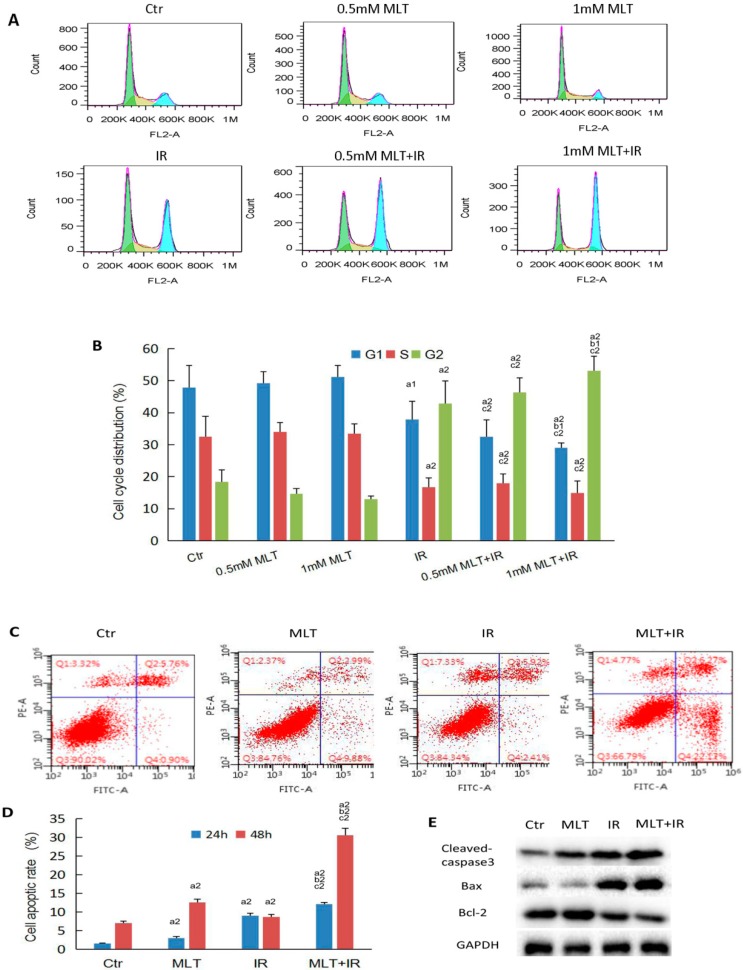
Melatonin-induced cell cycle redistribution and promoted apoptosis of the HCT 116 cells exposed to γ-ray radiation. (**A**) HCT 116 cells were treated with 0.5 mM or 1 mM melatonin for 2 h, then exposed to 6 Gy γ-ray radiation or not. The cell cycle distribution was examined after 24 treatment by flow cytometry. Representative images of cell cycle distribution are displayed; (**B**) the cell cycle distribution of HCT 116 was determined; (**C**) HCT 116 cells were treated with or without 1 mM melatonin for 2 h, then exposed to 6 Gy γ-ray radiation or not. The cell apoptosis was examined after 24 or 48 h treatment by flow cytometry. Representative images of cell apoptosis are displayed. Left lower quadrant denotes living cells, left upper quadrant denotes necrotic cells, right upper quadrant denotes late apoptotic cells, and right lower quadrant denotes early apoptotic cells; (**D**) the percentage of apoptotic cells was determined. Data are presented as the mean ± SD; (**E**) total protein was extracted after 2 h treatment and the levels of pro-apoptotic proteins, cleaved-caspase-3, Bax and anti-apoptotic protein Bcl-2 were detected by Western blot analysis. ^a1^ *p* < 0.05; ^a2^ *p* < 0.01 vs. control, ^b1^ *p* < 0.05; ^b2^ *p* < 0.01 vs. IR, ^c2^ *p* < 0.01 vs. MLT.

To investigate the mechanism behind the increased sensitivity to IR in HCT 116 cells treated with melatonin, we analyzed cell cycle distribution and cell apoptosis by flow cytometry. As shown in Figure 3B, the majority of control cells or melatonin-treated cells were blocked in the G1 phase before IR. However, combination treatment induced a higher proportion of cells in the G2 phase and simultaneously a decrease in the percentage of cells in the G1 phase and the S phase compared with the control or melatonin alone. Cell apoptosis is one of the important determinant of radiosensitivity. As shown in flow-based images of cell apoptosis (Figure 3C), the percentage of apoptotic cells (including early apoptotic cells and late apoptotic cells) of the IR group or melatonin group was increased after 24 or 48 h treatment compared with the control, and apoptotic cells were significantly increased after treatment with melatonin plus IR compared to cells treated with melatonin or IR alone (Figure 3D).

Caspases family plays a central role in the execution phase of cell apoptosis. As an executioner caspase, the caspase-3 zymogen is cleaved by an initiator caspase after apoptotic signaling events have occurred, which finally results in cell apoptotic. We investigated the expression of apoptotic-related proteins by Western blot analysis. It was found that the level of cleaved-caspase-3 and pro-apoptotic protein Bax were increased while the anti-apoptotic protein Bcl-2 was decreased in the combination treatment cells compared with those in single melatonin or IR treatment cells (Figure 3E). These cell apoptotic results from flow cytometry and Western blot analysis were consistent and indicated that melatonin had a pro-apoptotic effect on HCT 116 cells following radiation.

### 2.4. Effect of Melatonin on DNA Damage of HCT 116 Cells Induced by Radiation

**Figure 4:**
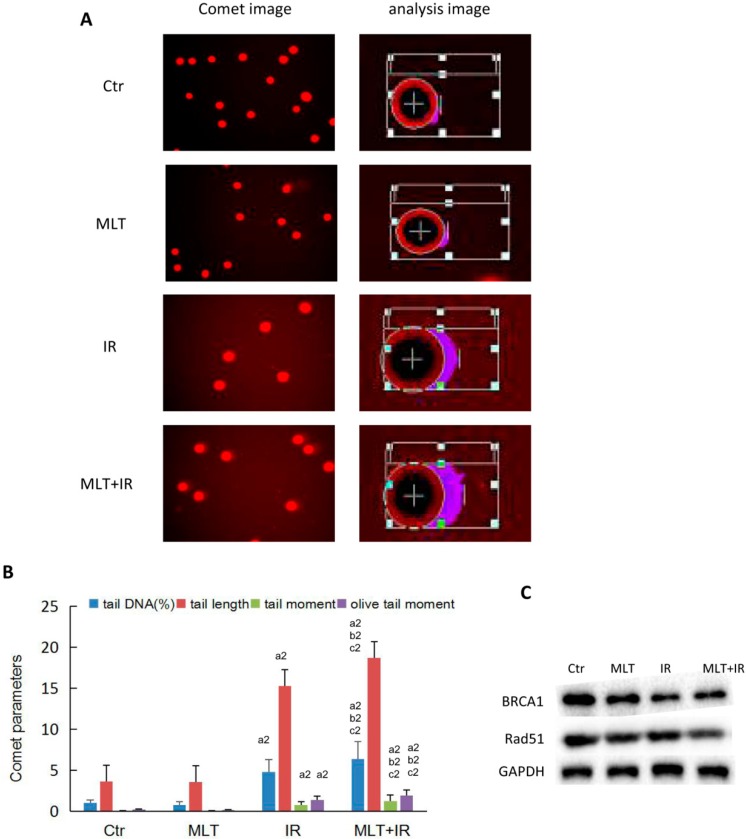
Melatonin accumulated DNA damage of HCT 116 cells exposed to γ-ray radiation. (**A**) HCT 116 cells were treated with or without 1 mM melatonin for 2 h, then exposed to 6 Gy γ-ray radiation or not. The comet assay was adopted to determine DNA damage after 2 h treatment. Representative images of comet assay in HCT 116 cells are displayed; (**B**) the DNA percentage in the comet tail (tail DNA %), tail length, tail moment, and olive tail moment representing the degree of DNA damage was calculated by analyzing 200 cells/sample; (**C**) total protein was extracted after 2 h treatment and the levels of proteins related with DNA damage and repair were detected by Western blot analysis. Data are presented as the mean ± SD. ^a2^ *p* < 0.01 vs. control, ^b2^ *p* < 0.01 vs. IR, ^c2^ *p* < 0.01 vs. MLT.

To evaluate the influence of melatonin on DNA damage to colorectal carcinoma cells after radiation, comet assay was used to detect the levels of DNA strand breaks in HCT 116 cells. As shown in Figure 4A, IR caused obvious damage to DNA. Consistent with our prediction, the comet assay showed that melatonin combined with IR induced a higher percent of tail DNA, tail length, tail moment, and olive tail moment than IR or melatonin alone (Figure 4B).

Moreover, we detected the level of proteins involved with DNA damage and damage repair in HCT 116 cells by Western blot analysis. As shown in Figure 4C, the expression of Rad51 and BRCA1 proteins was mildly lower in IR or melatonin treatment cells compared with those in the control cells, while melatonin plus IR treatment resulted in a marked decrease of Rad51 and BRCA1 expression compared with melatonin or the control. These results indicated that the combination of melatonin and IR treatment might decrease DNA repair efficiency.

### 2.5. Effect of Melatonin on Tumorigenicity in Nude Mice Induced by Radiation

**Figure 5:**
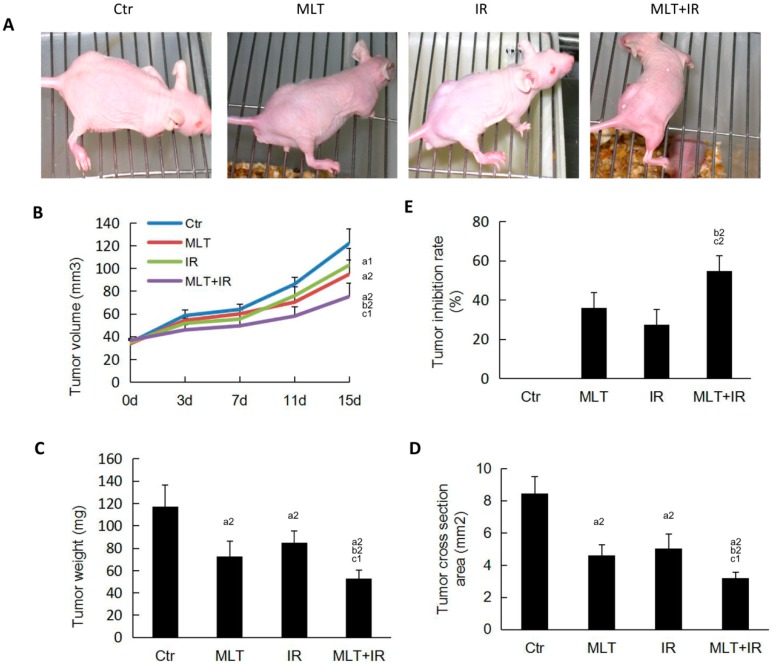
Melatonin suppressed tumor growth in nude mice exposed to γ-ray radiation. (**A**) The effect of melatonin and/or IR on tumorigenicity was examined in nude mice. Mice were treated with melatonin, IR, or a combination of melatonin and IR when tumor volume reached 40 mm^3^. Photographs of the tumor-bearing mice are displayed; (**B**) tumor growth curve was determined; (**C**) tumor weight was examined after mice were sacrificed at 15 days; (**D**) tumor sections were stained with H.E. and tumor cross-section area was calculated after mice were sacrificed at 15 days. (E) Tumor inhibition rate of xenografts at day 15 was analyzed. Tumor inhibition rate = (Weight_control group_ – Weight_treated group_)/Weight _control group_ × 100%. Data are presented as the mean ± SD. ^a1^ *p* < 0.05; ^a2^ *p* < 0.01 vs. control, ^b2^ *p* < 0.01 vs. IR, ^c1^ *p* < 0.05; ^c2^ *p* < 0.01 vs. MLT.

To determine the effect of treatment with melatonin and/or IR on tumor growth in vivo, a xenograft model was performed in nude mice. As shown in Figure 5B, the tumors of the control group grew abruptly from day 7, while that of the melatonin and IR group grew slowly. In addition, the tumor volume of the combination treatment group was decreased significantly compared with that of melatonin and IR group. Mice were sacrificed at day 15, and the data of tumor weight (Figure 5C) and tumor cross section (Figure 5D) showed that combination treatment has better efficacy in inhibiting tumor cell growth than that of melatonin or IR alone. Consistently, the tumor inhibition rate of melatonin treatment prior to IR was higher than melatonin or IR alone (Figure 5E). Taken together, melatonin rendered tumor xenograft more sensitive to IR and thus suppressed the growth of xenografts.

## 3. Discussion

In the current study, we firstly evaluated the effect of melatonin on the cell viability of human HCT 116 colorectal cancer cells in vitro. The result demonstrated that melatonin effectively inhibited HCT 16 cell proliferation (Figure 1). Then, we investigated the effects of the combination of melatonin and IR on HCT 116 cells in vitro. It was found that pretreatment of HCT 116 cells with melatonin before IR led to a significantly high decrease of colony formation and cell migration and a high increase of cell apoptosis and DNA damages compared with melatonin or IR alone (Figure 2, Figure 3 and Figure 4). Consistently, tumor xenograft studies showed that the combination treatment of melatonin and IR retarded the tumor growth more effectively than melatonin or IR alone in vivo (Figure 5). Collectively, our data suggested that melatonin improved the sensitivity of colorectal cancer to IR in vitro and in vivo.

To explore the mechanism by which melatonin enhanced anti-tumor efficiency following radiation, the cell cycle distribution, cell apoptosis and DNA damage assays were investigated. Cells in certain cycle phases are of different radiosensitivity, with the G2/M phase cells being most radiosensitive, the G1 phase cells less sensitive, and the latter part of the S phase cells least sensitive [19]. Hong et al. [18] reported that melatonin treatment activated cell death and led to G1 phase arrest at the advanced phase of HCT116 colorectal cancer cells. Liu et al. [26] revealed that melatonin promoted apoptosis of murine gastric cancer cells and induced cell cycle arrest at the G2/M phase. Here, our results showed that the concomitant use of melatonin and IR resulted in a significant cell cycle arrest in the G2/M phase with a lower proportion of cells in the G1 phase and S phase (Figure 3B), which was consistent with Liu’s findings. As cells arrested in the G2/M phase have greater sensitivity to IR than those in other phases [27], it is plausible that the increased proportion of cells arrested in the G2/M phase induced by melatonin treatment before IR may contribute to the decrease in HCT116 cell proliferation.

DNA is the most crucial target for the toxic effects of radiation. The most lethal type of DNA lesion induced by IR is DNA double-strand break (DSB). DNA damage and repair efficiency is one of the most critical factors determining the radiosensitization or chemosensitization in therapy for cancer. An increase in DNA repair capacity may induce the resistance of tumor therapy [28]. In mammalian cells, homologous recombination (HR) error-free repair pathway is the main mechanism for the repair of DSB. Increased levels of Rad51 protein in tumor cells plays a central role in the HR-mediating repair of DSB [29]. BRCA1 was reported to interact with RAD51 to repair DSB in the HR pathway [30]. A previous study indicated that melatonin sensitized human breast cancer cells to IR by downregulating the expression of RAD51 and DNA PKcs, proteins involved in DSB repair, compared to irradiated cells only [31]. In addition, melatonin decreased the effectiveness of DNA repair and increased the amount of DNA damage in the clinical treatment of non-small-cell lung cancer and colorectal adenocarcinoma cell lines [32]. Consistent with the above studies, we found that the combination of melatonin and IR led to a significantly high decrease in RAD51 and BRCA1 protein expression compared with melatonin or IR alone (Figure 4C), which reduced the repair of IR-induced DSB and caused more DNA damage (Figure 4B). Thus, colorectal cancer cells treated with melatonin conferred sensitivity to IR. 

Cancer treatment by radiotherapy or chemotherapy kills target cells primarily by inducing apoptosis. The most common regulatory genes involved in apoptosis are the members of B-cell lymphoma 2 (Bcl-2) family, which induce (pro-apoptotic) or inhibit (anti-apoptotic) apoptosis. Bcl-2-associated X (Bax) acts as a pro-apoptotic regulator that is involved in p53-mediated apoptosis, while Bcl-2 acts as an anti-apoptotic regulator. Previous studies revealed that melatonin enhanced antitumour effects in human gastric carcinoma cells [33] and murine foregastric carcinoma cells [34] by increasing Bax expression and decreasing Bcl-2 expression. Apoptosis is characterized by the activation of executioner caspases related to cell viability [35]. The pro-apoptotic effect of melatonin has been found in HT-29 human colon cancer cells by increasing caspase-3-type activity and DNA fragmentation [36]. Consistent with these reports, we found that melatonin pretreatment before IR resulted in significantly increased levels of Bax and decreased levels of Bcl-2 compared with those treated with melatonin or IR alone (Figure 3E), which resulted in increased cleaved-caspase3 and induction of apoptosis (Figure 3D). Melatonin plus IR activated the caspase-dependent apoptotic pathway, suggesting that melatonin hindered the proliferation and thus enhanced the sensitivity of colorectal cancer cells to IR.

In order to determine its potential toxicity, studies in vitro and in vivo reported a wide range of melatonin doses (25–200 mg/kg) tested in animal tumor models [26,37]. It was found that the toxicity of melatonin was extremely low and no lethal dose for animals was defined. Randomized clinical trials indicated that oral supplementation of melatonin (20 mg/day) could improve the quality of life or minimize radiation-induced side effects in cancer patients [21,38]. It seems that melatonin may be an ideal candidate for use as an adjuvant in cancer therapies. In our study, melatonin was used at pharmacological concentrations up to 2 mM, which had nothing to do with physiological situations. Our results demonstrated that melatonin has radiosensitization effects on colorectal cancer, which can decrease the damage inflicted to the normal tissue. It was worth mentioning that in the research on melatonin’s radiation protection for bodies, we found melatonin administration to be protected against acute intestinal injury induced by abnominal irradiation. Therefore, melatonin may reinforce the therapeutic effects and reduce IR-induced side effects in radiation oncology treatment.

In summary, melatonin pretreatment before IR significantly sensitized colorectal cancer cells to the ionizing effects of radiation, as assayed in both in vitro cell culture and in vivo xenograft tumor models. Melatonin enhanced the radiosensitivity of colorectal cancer cells to IR via inducing cell cycle arrest in the G2/M phase, downregulating proteins involved in DSB repair and activating the caspase-dependent apoptotic pathway. Thus, melatonin may act as an effective radiation sensitizer against colorectal cancer and have great potential as an adjuvant therapy in the future. Further experiments and clinical trials on this subject are needed to fully confirm it.

## 4. Materials and Methods

### 4.1. Cell Culture and Drug Treatment

The human colon cancer cell line (HCT 116) was purchased from the Cell Culture Center of Basic Medicine, Chinese Academy of Medical Sciences, Beijing, China. Cells were cultured in IMDM culture media (HyClone, Beijing, China) and supplemented with 10% FBS and a 100 U/mL penicillin, 100 μg/mL streptomycin solution in a humidified 5% CO_2_ incubator at 37 °C. Melatonin was purchased from Sigma-Aldrich (St. Louis, MO, USA). Melatonin was dissolved in a small amount of absolute ethanol (50 μL) and then diluted with isotonic NaCl solution to give a final ethanol concentration of 10% (*w*/*v*). HCT116 cells were treated with different doses of melatonin (0–2 mM).

### 4.2. Animals

Male BALB/c nude mice were purchased from Beijing HFK Bioscience Co. Ltd. (Beijing, China). All mice were used at approximately 6–8 weeks of age. They were maintained under controlled laboratory conditions of temperature (23 ± 2 °C), humidity (50 ± 5%) and light (light/dark 10/14 h). The mice were housed in the certified animal facility at Institute of Radiation Medicine of Chinese Academy of Medical Science. The experimental protocol was approved by the Animal Care and Ethics Committee of the Institute of Radiation Medicine (approval number: DWLL-20180112), and was performed according to the principles of the Institutional Animal Care and Ethics Committee guidelines.

### 4.3. Ionizing Radiation

An irradiator equipped with a Cs-137 (Gammacell-40) source was purchased from Atomic Energy Co. (Atomic Energy of Canadian Inc., Mississauga, ON, Canada). Cells and mice were exposed to ionizing radiation (IR) in the irradiation chamber and exposed to the radioactive source, delivering uniform irradiation at a dose rate of 0.99 Gy·min^−1^.

### 4.4. MTT Assay

HCT 116 cells were seeded at a density of 4000/well in 96-well tissue culture plates and then treated with the concentrations of melatonin (0.1, 0.5, 1, and 2 mM), followed by a 24, 48, and 72 h incubation separately. To each well was added 20 μL methylthiazol-2-yl-2,5-diphynyl,tetrazolium bromide (MTT) (5 μg/mL, Amresco Inc., Solon, OH, USA), and then the wells were incubated for 4 h at 37 °C. Then supernatant was removed and 150 μL dimethyl sulphoxide (DMSO) was added. The absorbance of each well was measured by the Multi-Mode Microplate Reader (Synergy HT, BioTek, Winooski, VT, USA) at 570 nm.

### 4.5. Colony Formation Assay

HCT 116 cells were plated at a density of 1000 cells/well in 6-well tissue culture plates treated with 1 mM melatonin for 2 h, and exposed to 0–8 Gy of ^137^Cs γ-radiation. A group of sham-irradiated control cells was included as control. During the 2-week incubation, the culture medium was replaced every 3 days without any drug in all wells. Finally, the cells were stained with Giemsa staining, and colonies containing more than 50 cells were counted.

### 4.6. Wound Healing Assay

HCT 116 cells were plated at a density of 20,000 cells/well in 6-well tissue culture plates. When the cells formed a monolayer, a scratch was generated in the middle of the well with a 100-µL pipette tip. Subsequently, the debris was removed and the cells were cultured with fresh media with 1 mM concentration of melatonin for 2 h, and exposed to 6 Gy radiation. A group of sham-irradiated control cells was included as control. After incubation for 48 h, the cells were imaged using a phase-contrast microscope. The initial migration of the scratch in the field of view was determined by the number of cells migrating to the scratch using Image J software (Media Cybernetics Co., Bethesda, MD, USA). Results are expressed as the difference in migration cell numbers between 0 h and 48 h of treatment.

### 4.7. Single Cell Gel Assay (Comet Assay)

Single-cell gel electrophoresis (SCGE) was applied under alkaline conditions. HCT 116 cells were digested and harvested immediately, and resuspended in PBS at a concentration of 4–5 × 10^5^/mL; 500 μL of 0.75% normal-melting-point agarose was coated on the comet slides. When the first agarose layer was coagulated, 30 μL of cell suspension was mixed with 70 μL of 0.75% low-melting agarose as the second layer and spread on the first layer of frosted slides. The slides were placed in cold alkalinelysis solution (2.5 M NaCl, 10 mM Tris base, 1% N-sodium lauryl sarcosinate, 30 mM Na2EDTA, 10% DMSO, 1% Triton X-100) for 2.5 h at 4 °C. Then, the slides were immersed in cold alkaline electrophoresis buffer (pH = 10) for 20 min at 4 °C, and electrophoresis was performed at 30 V for 20 min in a horizontal electrophoresis tank. The slides were washed twice with PBS and stained with ethidium bromide (2.5 µg/mL). Finally, the slides were visualized with an ECLIPSE 90 i fluorescence microscope (Nikon, Tokyo, Japan) and comet images were analysed using the Comet Assay Software Project (CASP, Wroclaw University, Poland). DNA damages to HCT116 cells were described by the percentage of DNA in the comet tail (tail DNA%), tail length, tail moment, and olive tail moment.

### 4.8. Cell Cycle

HCT 116 cells were seeded at a density of 1 × 10^5^ cells/well in 6-well tissue culture plates. After cells adhered, samples were treated with 0.5 mM or 1 mM concentration of melatonin and/or 6 Gy irradiation and cultured for 24 h. Cells were harvested by trypsinization, fixed in 70% pre-chilled ethanol overnight, and resuspended in propidium iodide solution (50 μg/mL propidium iodide, 0.1% Triton X-100, and 0.1% sodium citrate in PBS) for 30 min at 4 °C in the dark. Data from ≥10,000 cells were analyzed by flow cytometer (BriCyte E6, Mindray, Shenzhen, China), and the percentage of cells in each phase of cell cycle was calculated by FlowJo software.

### 4.9. Cell Apoptosis

HCT 116 cells were seeded at a density of 1 × 10^5^ cells/well in 6-well tissue culture plates. After cells adhered, samples were treated with 1 mM concentration of melatonin and/or 6 Gy irradiation and cultured for 24 h and 48 h. Cells were harvested by trypsinization and incubated in 0.5 mL of binding buffer containing 0.5 μg/mL Annexin-V-FITC and 5 μg/mL propidium iodide for 30 min in the dark according to the protocol of FITC Annexin V apoptosis detection kit (BD pharmingen, San Diego, CA, USA). Propidium iodide-negative and Annexin-V-FITC-positive cells were considered as the apoptotic population. The apoptotic cells were measured by flow cytometer (BriCyte E6, Mindray, Shenzhen, China), and the percentage of apoptotic cells was analyzed by FlowJo software.

### 4.10. Western Blot Analysis

HCT 116 cells were lysed in RIPA buffer to obtain total protein, and total protein concentration was quantified using a Bicinchoninic acid (BCA) protein assay kit (Beyotime Biotechnology, Shanghai, China). An equal amount of total protein (20 μg) was subjected to 10% (*w*/*v*) SDS-PAGE gel and transferred onto polyvinylidene difluoride (PVDF) membranes (Millipore, Billerica, MA, USA). These membranes were blocked with 5% (*w*/*v*) milk and 0.1% (*w*/*v*) Tween 20 in Tris-buffered saline, and incubated overnight at 4 °C with primary antibody. Then, the appropriate horseradish peroxide-conjugated secondary antibody (Proteintech, Wuhan, China) was added on the membranes at room temperature. Finally, the proteins were detected with enhanced chemiluminescent substrate (Proteintech, Wuhan, China). The primary antibodies used are as follows: The anti-cleaved-caspase3, anti-Bax, anti-Bcl-2, anti-Rad51, anti-BRCA1 and anti-GAPDH antibodies were obtained from Cell Signaling Technology (Danvers, MA, USA).

### 4.11. Tumorigenicity in Nude Mice

Mice were injected subcutaneously into the right feet with 1.0 × 10^7^ HCT 116 cells suspended in 200 μL IMDM medium. When xenografts reached a mean volume of 40 mm^3^, mice were randomly divided into four groups (n = 6/group): (1) Control, mice of this group did not receive any treatment; (2) IR alone, mice were exposed to IR at a dose of 2 Gy at days 0, 2 and 4; (3) MLT alone, mice were given an intraperitoneal injection of melatonin (10 mg/kg body weight) once daily at days 0, 1 and 2; and (4) MLT+IR, mice were administered an intraperitoneal injection of melatonin (10 mg/kg body weight) once daily at days 0, 1 and 2. One hour after the final injections, mice were exposed to IR at a dose of 2 Gy at days 2, 4 and 6. Mice were anaesthetized using chloral hydrate solution (0.3 g/kg) intraperitoneally before radiation and delivered to the tumor area with the rest of the mice shielded. Tumors were measured over the skin in two dimensions with calipers at days 0, 3, 7, 11, and 15. Tumor volumes were calculated using the formula: V (mm^3^) =1/6 π × length (mm) × width^2^ (mm^2^). Mice were sacrificed at day 15 and tumor mass was measured in three dimensions, dissected and weighed.

### 4.12. Statistical Analysis

All experiments were repeated at least two times, and results are presented as means ± standard deviation (SD). The Student’s *t* test was applied for statistical comparisons between groups. Statistical significance was considered at *p*-values < 0.05.

## Abbreviations

MLT	Melatonin
IR	Ionizing radiation
DSB	DNA double-strand break
HR	Homologous recombination
Bcl-2	B-cell lymphoma 2
Bax	Bcl-2-associated X
MTT	Methylthiazol-2-yl-2,5-diphynyl,tetrazolium bromide
SCGE	Single-cell gel electrophoresis
